# What Instagram Can Teach Us About Bird Photography: The Most Photogenic Bird and Color Preferences

**DOI:** 10.1177/20416695211003585

**Published:** 2021-04-22

**Authors:** Katja Thömmes, Gregor Hayn-Leichsenring

**Affiliations:** Cognitive Psychology Group, Department of Psychology, University of Konstanz, Germany; Experimental Aesthetics Group, Institute of Anatomy I, University of Jena, Germany

**Keywords:** Instagram data, photography, empirical aesthetics, color preferences

## Abstract

What makes a great bird photo? To examine this question, we collected over 20,000 photos of birds from the photo-sharing platform Instagram with their corresponding liking data. We standardized the total numbers of Likes and extracted information from the image captions. With this database, we investigated content-related image properties to see how they affect the ubiquitous online behavior of pressing a Like button. We found substantial differences between bird families, with a surprising winner in the category “most instagrammable bird.” The colors of the depicted bird also significantly affected the liking behavior of the online community, replicating and generalizing previously found human color preferences to the realm of bird photography.

To the bird watchers, ornithologists, and all the people with bird tables on their balconies: This paper will provide you with some delightful insights about how the visual appearance of our little feathered friends affects their aesthetic appeal towards the human observer. It showcases a method to leverage data from the social media platform Instagram to make empirically sound statements about why some bird photographs are aesthetically more appealing to us than others.

We utilize a method developed by Thömmes and Hübner (2020) that is based on real-life data from Instagram. Their so-called IAA (Image Aesthetic Appeal) score is based on the liking behavior of the large, diverse, and multicultural Instagram community. In a nutshell, the idea is to compute a measure of aesthetic appeal based on Instagram Likes. The score normalizes absolute numbers of Likes for time and reach, that is, for how many people have presumably seen an image (ranging from 10,000 to 500,000 viewers per image for the accounts investigated in this study). IAA scores are percentages above or below the Like estimation of a model using these confounds.^1^ The score is positive for images that receive more Likes than expected given the image’s exposure to viewers, and negative for images where the opposite is the case (Thömmes & Hübner, 2020). The IAA score proved to be a reliable measure^2^ for repeatedly published photos and it was shown to be predictive for experimentally collected liking ratings and aesthetic preferences (Thömmes & Hübner, 2018, 2020). The rationale behind designating the measure *aesthetic appeal* score is that pressing the Like button on Instagram—at least for large accounts sharing professional and consistent photographic content with a large followership—can be seen as an aesthetic behavior motivated by the aesthetic appeal of the image itself. For the selected Instagram accounts, this behavior is executed by many thousands of people for every single image and this wealth of data can be used to investigate aesthetic universals. As a proxy for the aesthetic appeal of images, the IAA measure has been successfully applied to replicate the preference for curved over angular shapes in architecture, landscape, and dance photography. It was also used to investigate low-level image statistics and high-level content-related image properties (Thömmes & Hübner, 2020). In this short paper, we want to apply the IAA measure to a new genre of photographic content—namely, bird photography.

Instagram is home to numerous accounts dedicated to sharing a constant stream of bird photographs with several hundred thousand followers. We picked nine of the largest bird accounts and collected a total of 27,621 images with corresponding meta data. We calculated IAA scores for each photo and extracted the species of every depicted bird from the captions. After excluding images without bird classification, we ended up with 23,818 bird portraits.^3^ From there, we set out to award Instagram’s most aesthetically appealing bird focusing on different bird families and effects of the birds’ color.

Figure 1 illustrates a ranking of 116 bird families based on average IAA scores. The surprising winner in this ranking is the *frogmouth* which seems to be a matter of poetic justice, as this nocturnal bird with very distinct facial features was once designated “the world’s most unfortunate-looking bird” (Van Dyck, 2004). Other birds high up in the ranking are colorful *pigeons* with decorative plumage, the emerald *turaco* with its crown-like head feathers and the *hoopoe* also wearing a distinct feather crown and showing off typical high-contrast feathering. We find that Instagram users are rather indifferent towards photos of *munias* with an average IAA score close to zero. On the low end, we find two types of seabirds: the *sandpiper* and the *oystercatcher*, which are often caught in the act of eating lugworms and seashells. *Storks* and *vultures* complete the team of the not-so-pretty birds. The ranking of bird families demonstrates that the IAA score is not necessarily tied to the beauty of the depicted bird. Presumably, interestingness, idiosyncrasy, and the situational context all play their part in the aesthetic appeal of bird photos to the human observer.

**Figure 1.**
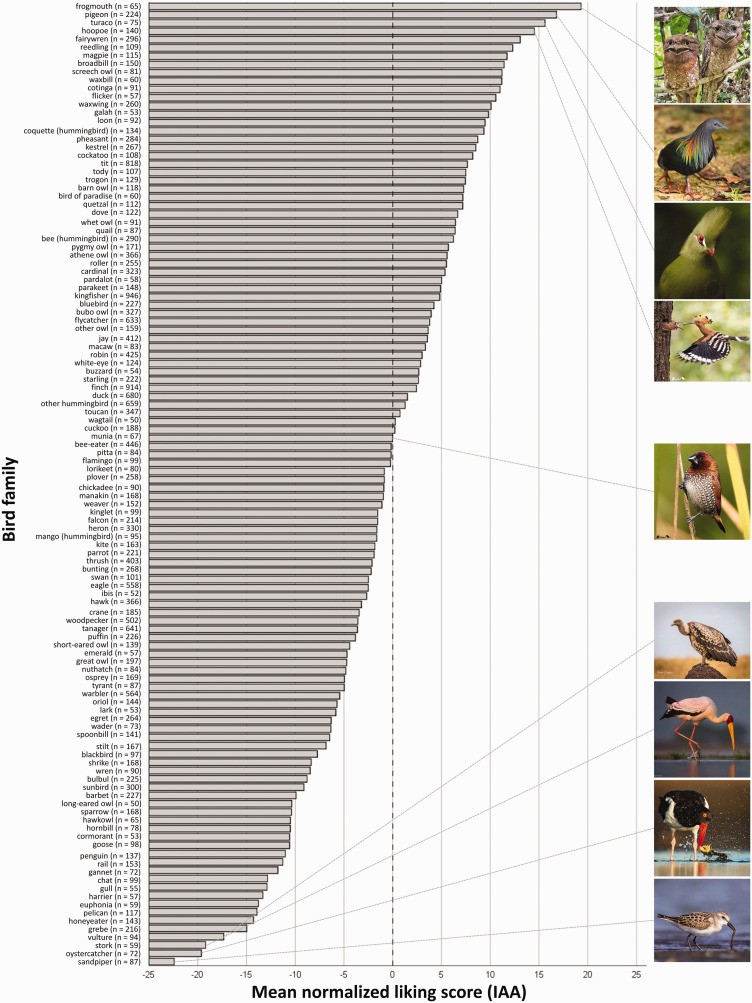
Bird family ranking. We analyzed 116 bird families with at least 50 images each and found substantial differences in mean IAAs (η^2^ = .045). The photos illustrate the four most and least liked birds, respectively, as well as the bird with the IAA score closest to zero.

In addition, we investigated effects of the bird’s color. There is a solid body of research on human color preferences indicating that blueish objects are generally preferred over objects with yellowish hues (right part of Figure 2). This has been explained by ecological valence, for example, blue being linked to good things such as clear sky and clean water, whereas potentially harmful objects such as rotten food are often yellow (Palmer & Schloss, 2010). We handpicked subsamples of brightly colored birds from the database and compared their mean IAA scores. The left part of Figure 2 illustrates how bird colors affect the IAA measure. Interestingly, the results closely correspond with the hypothesized color preferences that were previously reported for colored squares, but also for objects like furniture and clothing (Schloss et al., 2013).

**Figure 2.**
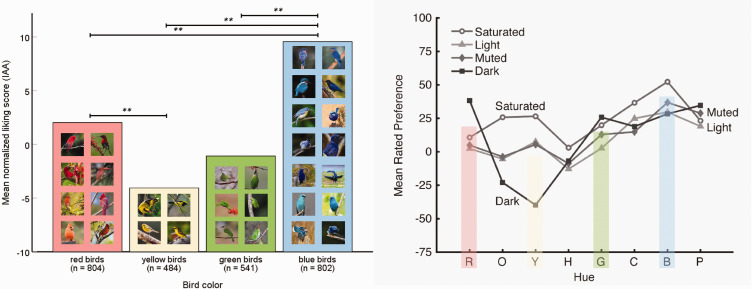
Left: Human bird color preferences (η^2^ = .028). **The mean difference is significant at the 0.01 level. Right: Human color preferences (modified after Schloss et al., 2013).

The reported effects illustrate the potential use of readily available Instagram data for aesthetic research. We consider this kind of data especially useful for the analyses of small effects where large datasets are necessary. Furthermore, Instagram users are intrinsically motivated to spend time on the app and interact with photos—and IAA scores are a by-product of such voluntary online behavior which might be advantageous over aesthetic ratings from lab experiments. With Instagram bird photography as an example of application, we hope to motivate future research to implement real-life data in empirical aesthetics.

## Notes

1 IAA scores are computed using the following equation: $\mathrm{IAA}=\frac{\mathrm{observed}\;\mathrm{Likes}\;-\;\mathrm{predicted}\;\mathrm{Likes}}{\mathrm{predicted}\;\mathrm{Likes}}\;\times 100$.

2 The authors report the intraclass correlation coefficient (ICC) to quantify test–retest reliability (ICC = 0.77).

3 We made the data available for further analysis here: osf.io/xng6q
